# Cisplatin-Induced Giant Cells Formation Is Involved in Chemoresistance of Melanoma Cells

**DOI:** 10.3390/ijms21217892

**Published:** 2020-10-24

**Authors:** Chien-Hui Weng, Chieh-Shan Wu, Jian-Ching Wu, Mei-Lang Kung, Ming-Hsiu Wu, Ming-Hong Tai

**Affiliations:** 1 Department of Biological Sciences, National Sun Yat-Sen University, Kaohsiung 80424, Taiwan; d952010003@student.nsysu.edu.tw; 2 Department of Dermatology, Kaohsiung Veterans General Hospital, Kaohsiung 81362, Taiwan; dermawu@vghks.gov.tw; 3 Department of Dermatology, Faculty of Medicine, College of Medicine, Kaohsiung Medical University, Kaohsiung 80708, Taiwan; 4 Biobank and Tissue Bank, Kaohsiung Chang Gung Memorial Hospital, Kaohsiung 83301, Taiwan; djbluestyle338@hotmail.com; 5 Department of Medical Education and Research, Kaohsiung Veterans General Hospital, Kaohsiung 81362, Taiwan; kungmeilang@gmail.com; 6 Department of Nutrition and Health Science, Fooyin University, Kaohsiung 83102, Taiwan; 7 Institute of Biomedical Sciences, National Sun Yat-Sen University, Kaohsiung 80424, Taiwan

**Keywords:** melanoma, chemoresistance, cisplatin, giant cell, stemness

## Abstract

Melanoma is notoriously resistant to current cancer therapy. However, the chemoresistance mechanism of melanoma remains unclear. The present study unveiled that chemotherapy drug cisplatin induced the formation of giant cells, which exhibited enlargement in cell diameter and nucleus in mice and human melanoma cells. Giant cells were positive with melanoma maker S100 and cancer stem cell markers including ABCB5 and CD133 in vitro and in vivo. Moreover, giant cells retained the mitotic ability with expression of proliferation marker Ki-67 and exhibited multiple drug resistance to doxorubicin and actinomycin D. The mitochondria genesis/activities and cellular ATP level were significantly elevated in giant cells, implicating the demand for energy supply. Application of metabolic blockers such as sodium azide or 2-deoxy glucose abolished the cisplatin-induced giant cells formation and expression of cancer stemness markers. The present study unveils a novel chemoresistance mechanism of melanoma cells via size alteration and the anti-neoplastic strategy by targeting giant cells.

## 1. Introduction

Melanoma, a malignant tumor of melanocytes, is much more dangerous than other skin cancers, because of its aggressive local growth and metastasis [1,2]. Melanoma has a high mortality and is notoriously resistant to all current modalities of cancer therapy [2,3]. Surgical resection is the most effective form of treatment during the initial phases of this disease. Although there are a variety of anticancer strategies, which range from surgery to immuno-, radio-, and chemotherapy, the average survival rate of therapy for melanoma is still 6 to 10 months [4].

The treatment of malignant melanoma has been limited by its broad resistance to chemotherapy. Cisplatin is one of the most potent and frequently used antitumor agents and is clinically effective against a variety of solid tumors, including ovarian carcinoma, adrenocortical carcinoma, and malignant melanoma [5]. Cisplatin is used in first- and second-line combination chemotherapy against melanoma [3]. The major cytotoxic effect of cisplatin is mediated by its interaction with DNA to form platinum-DNA adducts, which leads to DNA damage and induction of apoptosis [6]. The reduction in intracellular drug accumulation plays a major mechanism of acquired resistance to cisplatin in cancer [7,8,9]. However, the mechanism of tumor resistance to cisplatin is still not completely understood. Therefore, it is important to elucidate the mechanism(s) for cisplatin resistance to improve its therapeutic index and overcome the resistance of cancer cells to this therapy.

The cancer chemotherapeutic efficacy is usually limited by tumor resistance. The phenomenon of cancer cells with intrinsic or acquired drug resistance is termed multidrug resistance (MDR) [10]. Membrane transporters play key roles that affect drug entry into and extrusion from these cells, and are associated with chemotherapy efficiency [7,10,11]. P-glycoprotein (P-gp) is an ATP-dependent drug efflux transporter that belongs to the ATP-binding cassette (ABC) superfamily, which mediates multidrug resistance in mammalian cancer. The expression of ABC multidrug transporters has been implicated in the tumor cell development of drug resistance to anticancer therapy [11]. A novel human ABC transporter, ABCB5 (ATP-binding cassette, sub-family B (MDR/TAP), member 5), has been reported to be expressed significantly more in primary and metastatic melanoma than in benign melanocytic nevi; moreover, the expression of ABCB5 is higher in metastatic melanomas than in primary lesions [12,13]. The malignancy of a tumor is another important factor that helps determine the mortality and efficiency of an anticancer drug. There are several proteins associated with the malignancy of melanoma in particular, such as S100, CD133, and ABCB5. S100 is involved in the regulation of several cellular processes, including cell growth, differentiation, cell motility, and the control of the cytoskeleton assembly [14]. S100 is one of the most sensitive markers for melanocytic malignancy [15,16]. CD133, is prominently located on the cell membrane protrusions, has been described as a cancer stem cell marker, and is one of most studied stem cell markers that are expressed by melanoma cells [17,18,19].

In previous study, atomic force microscopy (AFM) was used to record the biomechanical change of cisplatin-induced enlarged B16-F10 melanoma cells [20]. Our study unveiled that a certain portion of viable B16-F10 cells after cisplatin treatment showed significant increment in cell size/area and nucleus. We termed these cells as “giant cells.” Giant cells expressed several unique morphological characters that were distinct from those expressed in the parental B16-F10 cells, including a wide and flat cytoplasm and increase in the mitochondria number and the mitochondrial membrane potential. The aims of this study are to address the cellular events that are involved in the genesis of giant cells and to elucidate the pathological significance of these cells, especially in the development of chemoresistance and cancer stemness.

## 2. Results

### 2.1. Cisplatin Therapy Elicited the Formation of Giant Cells in Mice

**Figure 1:**
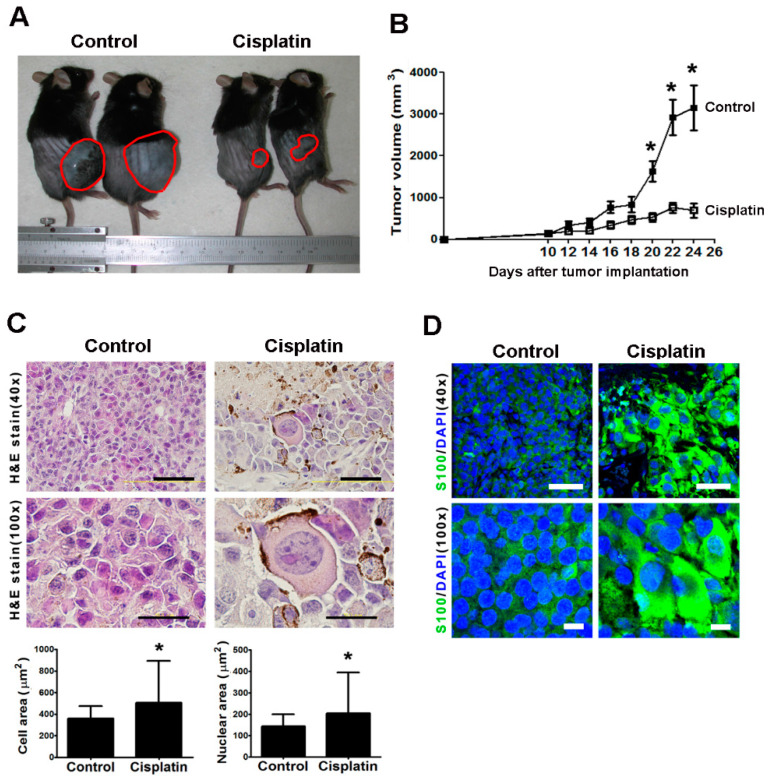
The existence of giant cells in cisplatin-treated B16-F10 melanoma in vivo. Tumors were induced through the subcutaneous transplantation of B16-F10 cells in C57/BL mice. (**A**) PBS-treated (control) and cisplatin-treated xenografted mice were anesthetized and shaved before being sacrificed. The red circles denote the tumor margins. (**B**) The tumor volumes in the mice (control group, *n* = 12; cisplatin-treated group, *n* = 12) were measured at the indicated time. (**C**) The tumors slices were stained with HE and observed by microscope with 40- and 100-fold magnifications. Scale bars, 100 µm and 50 µm at 40-fold and 100-fold magnifications, respectively. The areas of the cells and the nuclei were quantified using software (the measured cell number > 500, each group). (**D**) The tumor slices were immunostained by the anti-S100 antibody (green), and the nuclei were stained with DAPI (blue). Scale bars, 50 µm (top panel) and 10 µm (bottom panel). * *p* < 0.05 compared with the control group.

We studied the cellular morphological change of melanoma during chemoresistance in vivo. The B16-F10 cells grown in C57BL mice were treated with cisplatin therapy and then subjected to histological analysis. Cisplatin treatment significantly reduced the volume of the tumor by approximately 75% compared with the control group (Figure 1A,B). The quantification analysis showed that the mean cell and nuclear areas of the cisplatin-treated cells were 507 and 203 μm^2^, respectively, which were obviously higher than the respective areas of the control cells (360 and 143 μm^2^, respectively; Figure 1C). Through the HE and DAPI staining, we also observed that most of the nuclei were markedly larger in the cisplatin-treated group than in the control group (Figure 1C,D). Thus, we termed the enlarged B16-F10 cells as “giant cells” in vivo.

To study whether the cisplatin-induced giant cells are more malignant than their parental cells, we examined the expression of the protein S100. Cytoplasmic S100 was weakly expressed in the melanoma tissues of the control group and was more strongly expressed in the cisplatin-treated group (Figure 1D). These data indicate that the expression of the malignant melanoma maker S100 increased after cisplatin treatment, particularly in the giant cells. Thus, this result may have important implications for pathological diagnosis.

### 2.2. Cisplatin Induced the Formation of Giant Cells in Melanoma Cells In Vitro

**Figure 2:**
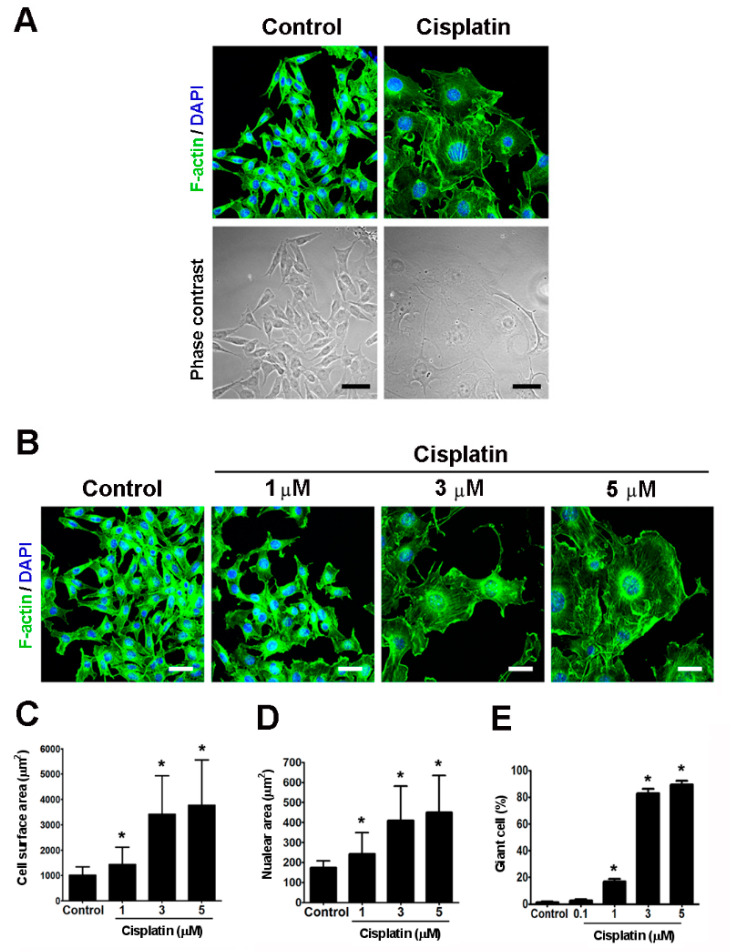
Cisplatin induced the formation of giant cells in vitro. (**A**) B16-F10 cells were treated with 3 µM cisplatin for 48 h and were stained with phalloidin to visualize the actin filaments (green), and the nuclei were stained with DAPI (blue). The fluorescence and phase contrast images were examined under the same field. Scale bar, 50 μm. (**B**) Cells were treated with different doses of cisplatin (1~5 µM) for 48 h. The fixed cells were stained with phalloidin (green) and the DAPI (blue). Scale bar, 50 µm. (**C**) and (**D**) The cell surface and nuclear areas were determined by software (the measured cell number > 100, each group). (**E**) The percentage of giant cells was examined after 48 h exposure to 0.1 to 5 µM cisplatin. The counted cell number in each group was > 300. * *p* < 0.01 compared with the control cells.

To investigate the characteristics of cisplatin-induced enlarged cells, B16-F10 cells were treated with cisplatin and subjected to confocal microscopy. Through phase contrast observation, it appeared that the cells were more transparent and flattened and exhibited an increased surface area after 48 h exposure to cisplatin (Figure 2A). By using various doses of cisplatin, it was observed that the cisplatin-induced cell enlargement was dose-dependent (Figure 2B). We then measured the nuclear and cell surface areas of the melanoma cells by confocal microscopy for quantitative comparison. In control B16-F10 cells, the average cell surface and nuclear areas were 1080.0 μm^2^ (about 99% control cells were in the range of 600–2000) and 174.9 µm^2^ (about 99% control cells were in the range of 150–250), respectively (Figure 2C,D). Thus, we defined the enlarged B16-F10 cells with surface areas over 2000 µm^2^ and nuclear areas over 250 µm^2^ as “giant cells” in vitro. The cisplatin-elicited increment in cell surface and nuclear areas were dose-dependent (Figure 2C,D). In addition, there was a significant correlation between giant cells formation and cisplatin dosage (Figure 2E). Moreover, there were about 1% spontaneous giant cells existed in control B16-F10 cells (Figure 2E). It was observed that treatment of B16-F10 cells with cisplatin at 3 µM led to 83 ± 5.7% of giant cells after 48 h, which was employed as the optimal condition for the induction of giant cells in the subsequent studies.

### 2.3. Giant Cells Exhibited Enlarged Volumes and Nuclei and Reduced Thicknesses and Motilities

**Figure 3:**
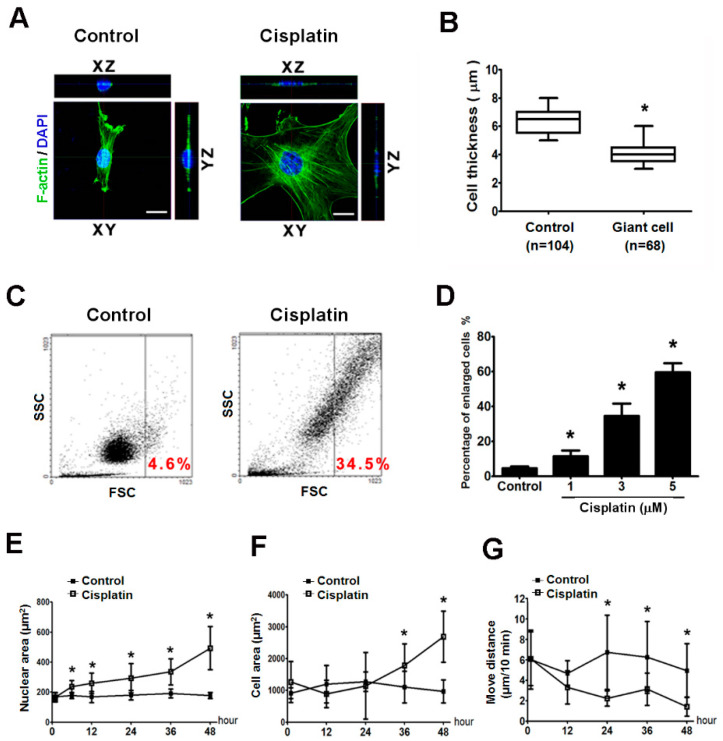
The characteristics of cisplatin-induced giant cells in vitro. B16-F10 cells were treated with 3 μM cisplatin for 48 h. (**A**) The 3D images were established by XZ and YZ cross sections of cells. Scale bar, 20 μm. (**B**) The thickness of the melanoma cells was measured by Z-stack confocal microscopy, and the results are presented as a box plot that illustrates the variation in the cellular thickness. * *p* < 0.05 compared with the control cells. (**C**) The cisplatin-induced enlarged cells were analyzed by flow cytometry. The forward scatter (FSC) represents the cell size (X axis), and the side scatter (SSC) represents the inner complexity of the cell (Y axis). The cell size was gated in the dot plot and defined the cisplatin-induced enlarged cells by the cell size. The percentages of enlarged cells indicated on the panel are the means of three independent experiments. (**D**) The percentages of enlarged cells induced by various doses of cisplatin are assayed by flow cytometry. * *p* < 0.05 compared with the control cells. (**E**,**F**) The nuclear and cell surface areas were determined by software. (**G**) The motilities of the cells were measured by the distance that a single cell travelled in 10 min. (**E**–**G**) * *p* < 0.05 compared with the control cells.

The thickness of the cells and organelles was also measured by Z-stack section analysis, and elicited a dramatic remodeling of actin network in melanoma cells (Figure 3A). The results are shown as box plots that statistically illustrate the variations in the cell thickness (Figure 3B). Although the cell surface area of the giant cells that were attached to the plate was increased, the thickness of the nuclei and cytoplasm of the giant cells was decreased. We further applied flow cytometry to monitor the cell size, and the cell size (FSC) was gated in the dot plot and defined the cisplatin-induced enlarged cells by the cell size. About 34.5% of the population was gated as enlarged cells after 3 μM cisplatin treatment compared with the volume of the control cells (Figure 3C). By measurement of flow cytometry, we found that cisplatin increased the ratio of enlarged cells in a dose-dependent manner (Figure 3D).

The time-lapse imaging captured the process of giant cell formation, which clearly showed the significant morphological changes that occur during the formation of giant cells (Supplementary Information: Videos S1 and S2). Cisplatin induced the increase of not only the cytoplasm but also the area of the nucleus (Figure 3E). The nuclei were significantly larger after 6 h exposure to cisplatin. In parallel, the whole cell area was significantly elevated after 36 h cisplatin treatment (Figure 3F). These data reveal that the area of nuclei increased earlier than the whole cell area during cisplatin treatment. According to time-lapse imaging, the cell motility decreased after cisplatin treatment (Figure 3G, Supplementary Information: Videos S1 and S2).

### 2.4. Giant Cells Expressed the Cancer Stemness Markers ABCB5 and CD133 In Vitro and In Vivo

**Figure 4:**
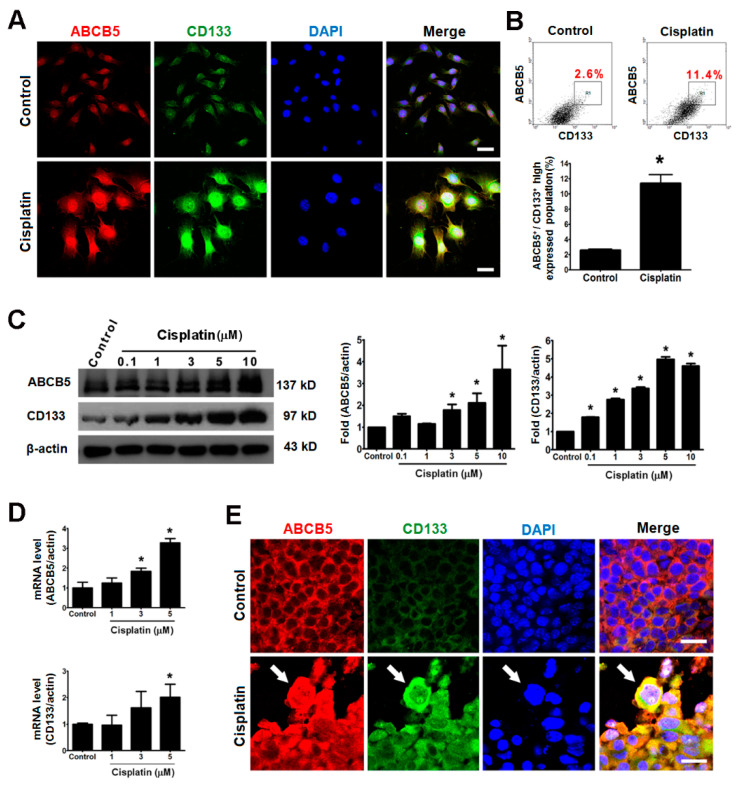
The expressions of ABCB5 and CD133 were elevated in the cisplatin-induced giant cells in vitro and in vivo. The in vitro evidence was obtained from B16-F10 cells that were treated with cisplatin (3 µM) for 48 h. (**A**) The immunofluorescence of ABCB5 (red) and CD133 (green) in the control B16-F10 cells (top panel) and the cisplatin-induced giant cells (bottom panel). The nuclei were stained with DAPI (blue), and the ABCB5 and CD133 signals that were merged (yellow) to indicate the co-expression of ABCB5 and CD133. Scale bar, 50 µm. (**B**) The expression of ABCB5 and CD133 were quantified by flow cytometry, and the population overexpressed both ABCB5 and CD133 was gated and shown in the dot plot, and statistical bar chart (bottom panel). The percentages of ABCB5^+^/CD133^+^ high expressed population indicated on the panel are the means of three independent experiments. (**C**) The translational levels of ABCB5 and CD133 were analyzed by Western blot by antibodies specific for ABCB5, CD133, and β-actin. The histograms show the proteins levels quantified by the ABCB5/β-actin and CD133/β-actin immunoband intensities. (**D**) The transcriptional levels of ABCB5 and CD133 were measured by qRT-PCR. (**E**) Images of immunofluorescence show that the giant cells overexpress ABCB5 (red) and CD133 (green) in tissue specimens from mice. The nuclei were stained with DAPI (blue), and the ABCB5 and CD133 signals were merged (yellow), and the white arrows indicate the giant cell with highly expressing ABCB5 and CD133. Scale bar, 20 µm. * *p* < 0.05 compared with the control group.

Immunofluorescent analysis revealed elevated ABCB5 and CD133 expressions in cisplatin-induced giant cells (Figure 4A). This result was further supported by flow cytometry, which revealed an increased ratio of ABCB5^+^/CD133^+^ cells after cisplatin treatment (Figure 4B). Immunoblot and qRT-PCR analyses showed that cisplatin elicited the upregulation of ABCB5 and CD133 in a dose-dependent manner (Figure 4C) and at the transcriptional level (Figure 4D). An immunofluorescent analysis of the melanoma tissues unveiled a stronger immunostaining of ABCB5 and CD133 in the giant cells of the cisplatin-treated tumors (Figure 4E). These data indicate that ABCB5 and CD133 were over-expressed in the cisplatin-induced giant cells.

### 2.5. Giant Cells Retained Their Proliferating Potential but Were Not Senescence

**Figure 5:**
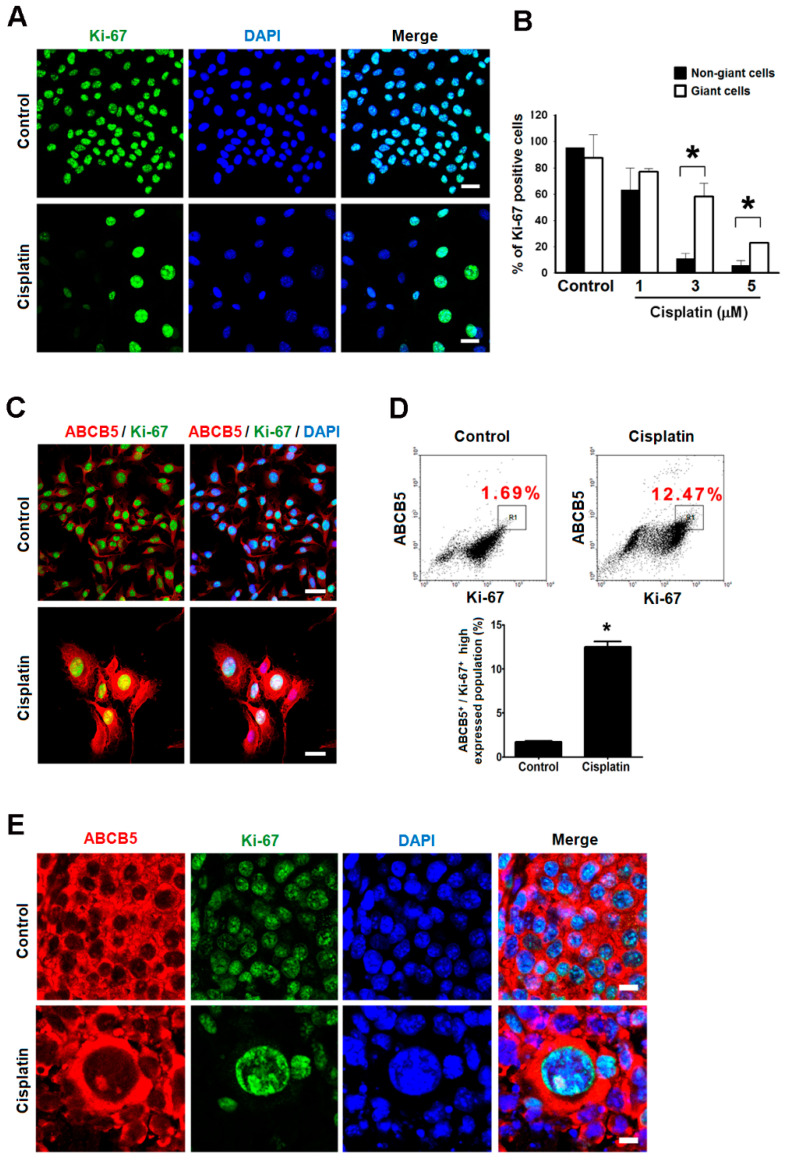
The expressions of Ki-67 and ABCB5 were detected by immunofluorescence after cisplatin treatment in vitro and in vivo. The proliferating maker Ki-67(green), ABCB5 (red) were co-immunostained with their respective antibodies; the nuclei were stained with DAPI (blue). (**A**,**C**,**D**) The in vitro evidence was obtained from B16-F10 cells that were treated with 3 µM cisplatin for 48 h. (**A**) The expression of Ki-67 was sustained in giant cells after cisplatin treatment. Scale bar, 50 µm. (**B**) The percentage of Ki-67 expression in the non-giant cells and the giant cells. * *p* < 0.05, compared with the non-giant cells treated with the same dose of cisplatin. (**C**) The co-expressions of Ki-67 and ABCB5 in control and cisplatin-treated B16-F10 cells. Scale bar, 50 µm. (**D**) The expressions of ABCB5 and Ki-67 were analyzed by flow cytometry (upper panel), and the top region of the ABCB5 and Ki-67 signals were gated for the quantification of the population that strongly co-express ABCB5 and Ki-67 (lower panel). The percentages of ABCB5^+^/Ki-67^+^ high expressed population indicated on the panel are the means of three independent experiments. * *p* < 0.01 compared with the control cells. (**E**) The cellular distribution of ABCB5 and Ki-67 in tissue specimens of control and cisplatin-treated tumor-burdened mice. Scale bar, 10 µm.

The proliferating potential of giant cells was characterized by the expression of Ki-67, a marker of cellular proliferation [21]. B16-F10 cells treated with various doses of cisplatin showed that 3 and 5 µM of cisplatin retained strong Ki-67 expression mainly in the giant cells but less in the non-giant cells (Figure 5A,B), and similar results were also observed from cisplatin-treated melanoma tissue specimen (Figure S1A). Because ABCB5 is a transport response to lower the accumulation of drugs [22], we hypothesized that the over-expression of ABCB5 reduces the cytotoxicity of cisplatin, which attenuated the cisplatin-induced growth arrest. Through immunofluorescent analysis, we found that cisplatin increased the population of ABCB5^+^/Ki-67^+^ in the giant cells (Figure 5C). This result was further validated by flow cytometry, which revealed significantly increased percentages of ABCB5^+^/Ki-67^+^ cells in cisplatin-treated cells compared with the control cells (Figure 5D). Despite of dramatic decrease in the number of Ki-67 positive population during cisplatin therapy, strong Ki-67 expression could still be detected in the giant cells with ABCB5 over-expression (Figure 5E). Interestingly, the division of giant cells could be observed in vitro and in vivo (Figure S1B,C and Movie S3). Since senescent cells also show morphologically flattened and enlarged cell shapes with senescence associated-β galactosidase (SA-gal) activity [23], we also determined the biomarker of cellular senescence by detection of SA-gal activity in giant cells. As shown in Figure S2A, most giant cells were negative for β-gal staining at 48 h after cisplatin treatment, indicating cisplatin-induced giant cells were not stress-induced cellular senescence. Because cellular senescence is the state of irreversible cellular growth arrest [24], these results show that certain giant cells retain the proliferating potential but were not senescence during cisplatin challenge.

### 2.6. Giant Cells Exhibited Enhanced Drug Resistance

**Figure 6:**
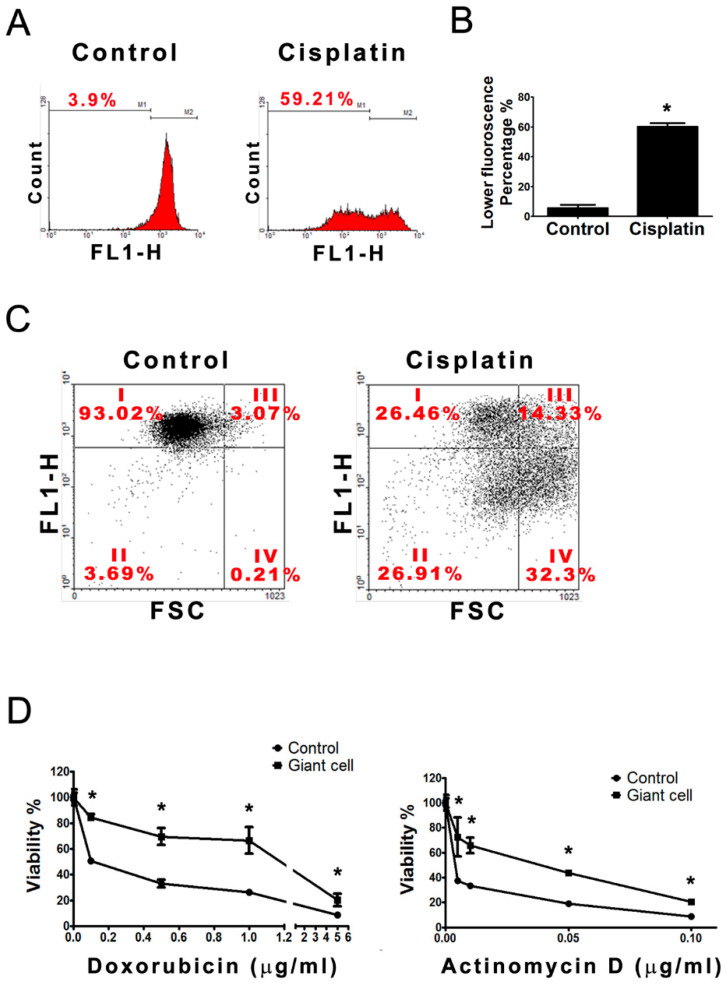
Cisplatin-induced giant cells exhibited chemoresistant properties. (**A**) After the formation of giant cells was induced through 48 h treatment with cisplatin, the chemoresistant activities were subsequently analyzed by multidrug resistance assays. The percentages represent the population with lower accumulation of calcein AM (FL1-H). (**B**) The data in the statistical bar chart are the flow cytometry results, and the less-fluorescent population represents the cells with a lower accumulation of the drug. * *p* < 0.01, compared with the control group. (**C**) The flow cytometric dot plots were further gated by the cell size (FSC) and the intensity of the calcein AM fluorescence (FL1-H). The region I represents the percentage of non-enlarged cells with higher accumulation of calcein AM; The region II represents the percentage of non-enlarged cells with lower accumulation of calcein AM; The region III represents the percentage of enlarged cells with higher accumulation of calcein AM; The region IV represents the percentage of enlarged cells with lower accumulation of calcein AM (**D**) The cellular susceptibilities of doxorubicin and actinomycin D were detected by crystal violet viability test in control cells and giant cells. The viability percentage of the untreated cells was considered to be 100%. * *p* < 0.01, compared with the control group at the same dosage.

Because ABCB5 was over-expressed in the cisplatin-induced giant cells (Figure 4 and Figure 5), we examined the drug resistance efficiency of the giant cells and found that cisplatin significantly elevated the percentage of drug-resistant cells to 59.21% of the cisplatin-treated cells compared with 3.9% in the control cells (Figure 6A,B), which suggests that the number of cells with multidrug resistance (MDR) increases after cisplatin treatment. Furthermore, the distribution of the population was changed after cisplatin treatment: the size of the cells increased and the drug accumulation decreased. As shown in Figure 6C, the population of enlarged cells with MDR ability (region IV) increased from 0.21% to 32.3%.

The drug sensitivity of giant cells against two antineoplastic drugs (actinomycin D and doxorubicin) was measured through a viability assay. B16-F10 cells were incubated with 3 µM cisplatin for 48 h to induce the formation of giant cells. Equal numbers of these cells were then re-plated and treated with various dosages of doxorubicin or actinomycin D. The viability assay showed that the IC50 of doxorubicin increased from 0.12 to 2.45 μg/mL and that the IC50 of actinomycin D increased from 4 to 40 ng/mL (Figure 6D). These results indicate that cisplatin-treated cells were cross-resistant to doxorubicin and actinomycin D.

### 2.7. Elevated Mitochondrial Activities and ATP Synthesis in Cisplatin-Induced Giant Cells

**Figure 7:**
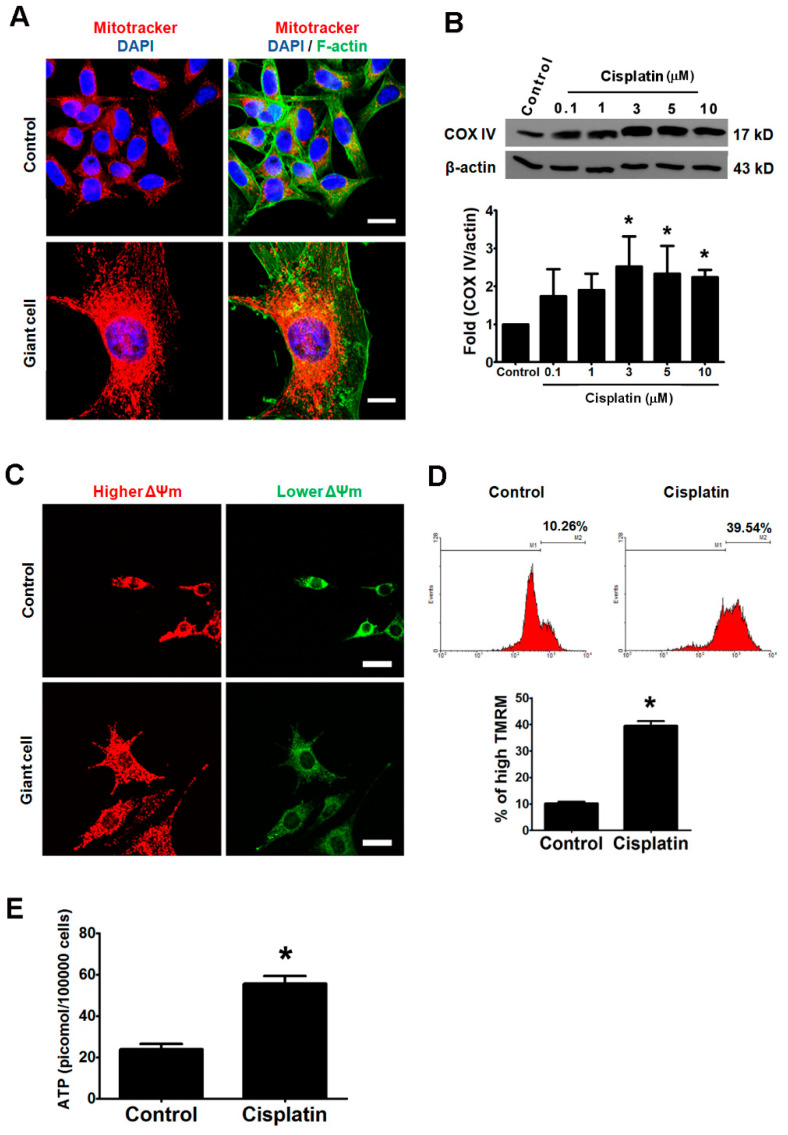
The cisplatin-induced giant cells exhibited high mitochondrial activities and high levels of ATP. (**A**) The formation of giant cells was induced by cisplatin in B16-F10 cells, and the amount of mitochondria was monitored. The control cells and the cisplatin-treated cells were co-stained with three dyes to analyze different cellular organelles: MitoTracker (red) for the mitochondria, phalloidin (green) for F-actin, and DAPI for the nuclei (blue). Scale bar, 20 µm. (**B**). The amounts of COX IV were increased in B16-F10 cells after 48-h treatment with different doses of cisplatin. The COX IV/β-actin immunoband intensities were determined by densitometric scanning. (**C**) The mitochondrial membrane potential (ΔΨm) was measured by using the JC-1 dye. A red color indicates a relatively high membrane potential, whereas a green color indicates a relatively low membrane potential. The giant cells displayed higher red fluorescence and lower green fluorescence. Scale bar, 50 µm. (**D**) The mitochondrial membrane potential was measured by TMRM dye, and was analyzed by flow cytometry (top panel; the X axis represents TMRM intensity; the Y axis represents cell count). The percentage of high TMRM population indicated on the panel are the means of three independent experiments. The bar chart displays the percentages of the population with a higher mitochondria membrane potential (bottom panel). (**E**) The cellular ATP of an equal number of control and cisplatin-treated cells was measured by a luciferin-luciferase bioluminescence method. * *p* < 0.01 compared with the control cells.

We found that the growth curve of cisplatin-treated B16-F10 cells assayed by counting the cell numbers was not correlated with parallel MTT assays (Figure S3). MTT test measures the viability by the activity of mitochondrial reductase [25], and therefore we speculate that the number and/or activity of the mitochondria are involved in the formation of giant cells. A mitochondria-selective dye (Mitotracker) was used to visualize the location and amount of mitochondria. The fluorescent intensity and amount of mitochondria increased in the giant cells compared with the control cells after 48 h cisplatin treatment (Figure 7A). To provide additional evidence, the expression of cytochrome c oxidase, also named complex IV (COX IV), was analyzed by immunoblot. COX IV, located in the mitochondrial membrane, and is associated with ATP synthesis. The cellular COX IV levels were elevated two- to three-fold after 48 h treatment with cisplatin, and these data indicate that the number of mitochondria increased after the treatment of cisplatin for 48 h (Figure 7B).

The activities of the mitochondria can be monitored by the mitochondrial membrane potential and the ability of ATP synthesis. The membrane potential-dependent JC-1 dye was used as an indicator of the mitochondrial membrane potential [26]. The red fluorescence (hyperpolarization) was increased and that the green fluorescence (depolarization) was decreased in the cisplatin-induced giant cells compared with the control cells (Figure 7C). The data suggest that the mitochondrial membrane potential was elevated in response to the formation of giant cells. To further quantify the cisplatin-induced variations in the mitochondrial membrane potential, tetramethylrhodamine methyl ester (TMRM), a cationic mitochondrial selective probe [27], was utilized to measure the mitochondrial membrane potential by flow cytometry. Compared with the control group, we found almost a four-fold increase in the populations with a higher TMRM signal in the cisplatin-treated group (Figure 7D). The cellular ATP levels increased approximately 2.3-fold compared with the control group after 48 h exposure to 3 μM cisplatin (Figure 7E). These data reveal that the cisplatin-induced giant cells contained increased numbers of mitochondria with a higher membrane potential and thus subsequently synthesize an increased amount of ATP.

### 2.8. Metabolic Blockers Abolished the Cisplatin-Induced Formation of Giant Cells and Expression of Cancer Stemness Markers

**Figure 8:**
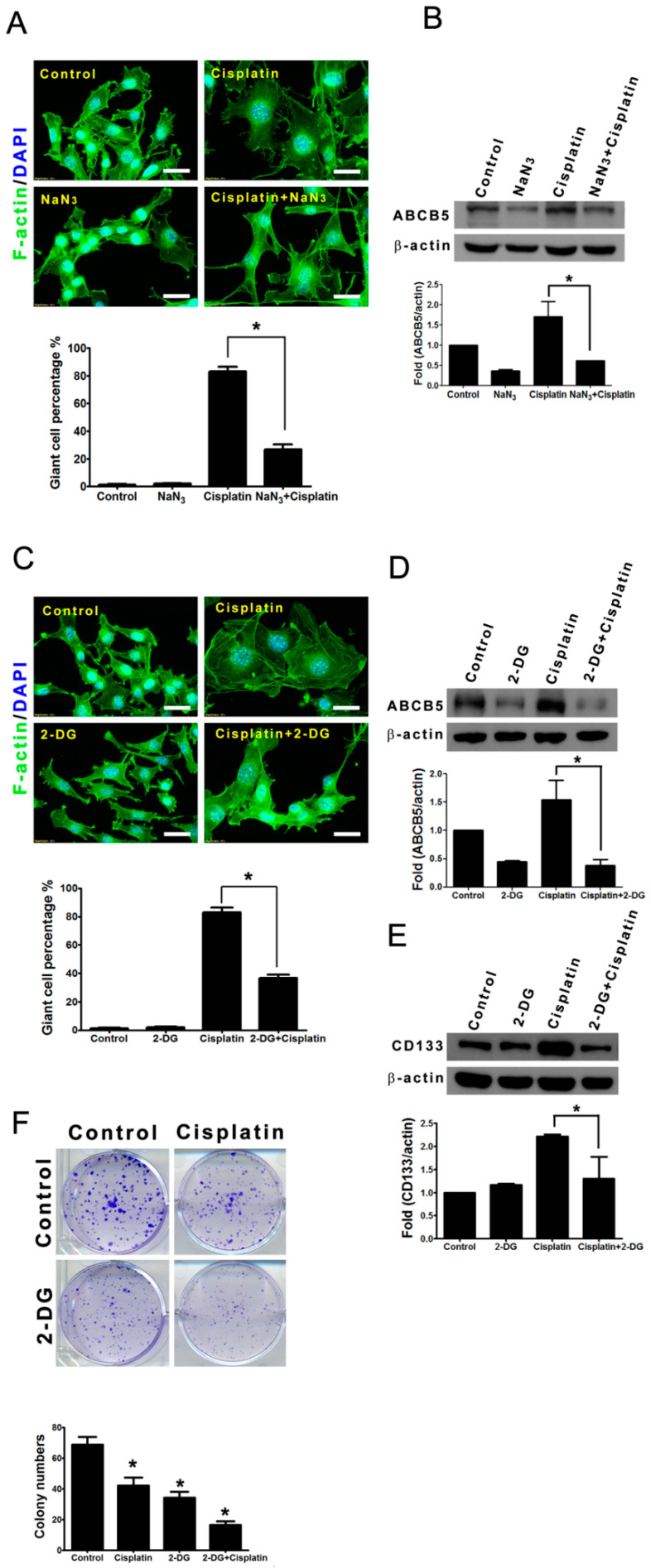
The cisplatin-induced formation of giant cells and expression of ABCB5 and CD133 were attenuated by sodium azide or 2-DG. B16-F10 cells were treated with 3 µM cisplatin in the absence or presence of sodium azide (NaN_3_) or 2-DG for 48 h. The cells were stained with phallotoxin (green) and DAPI (blue). Scale bar, 50 µm. The total cell lysates were analyzed by Western blot using antibodies specific for β-actin and either ABCB5 or CD133. The histograms show the protein levels of ABCB5 and CD133 quantified from the ABCB5/β-actin and CD133/β-actin immunoband intensities, respectively, that were determined through densitometric scanning. (**A**) The percentages of giant cells were calculated and are presented statistically in the bar chart. Scale bar, 50 µm. * *p* < 0.01, compared with the cisplatin-treated cells. (**B**) Sodium azide blocked cisplatin-induced expression of ABCB5. * *p* < 0.05, compared with the cisplatin-treated group. (**C**) The percentages of giant cells were calculated and are presented statistically in the bar chart. Scale bar, 50 µm. * *p* < 0.01 compared with the cisplatin-treated cells. (**D**,**E**) 2-DG blocked the cisplatin-induced expression of ABCB5 and CD133. * *p* < 0.05 compared with the cisplatin-treated group. (**F**) B16-F10 cells were treated with or without cisplatin (0.5 µM) in the absence or presence of 2-DG (1 mM) for 7 days to allow colony formation, and then stained with crystal violet. The bar chart displays the colony numbers in different treatment. * *p* < 0.05 compared with the control group.

Because the mitochondrial activities were elevated in the giant cells, we evaluated the influence of sodium azide, an inhibitor of the mitochondrial electron transport [28], on the cisplatin-induced formation of giant cells and cancer stemness. Treatment with 1 mM sodium azide potently perturbed the cisplatin-induced formation of giant cells and the cisplatin-induced expression of ABCB5 (Figure 8A,B). Similarly, we explored the role of ATP generation in the cisplatin-induced formation of giant cells. The glucose analog 2-deoxyglucose (2-DG), a glycolytic inhibitor, acts as a competitive inhibitor of the glucose metabolism and causes decrease of cellular ATP [29]. Treatment with 2 mM 2-DG for 48 h did not significantly cause cell death, and 2-DG treatment attenuated the cisplatin-induced formation of giant cells (decreased from 83% to 36%) (Figure 8C). Evidently, the percentage of giant cells was reduced by co-treatment with 2-DG and cisplatin, which indicate that a high amount of energy is required for the formation of giant cells. Interestingly, the cellular levels of ABCB5 and CD133 were also reduced by 2-DG (Figure 8D,E) implying that ATP was not only essential for the formation of giant cells but also required for ABCB5 and CD133 expression. Moreover, 1 mM 2-DG enhanced the cytotoxic effect of 0.5 µM cisplatin evaluated by colony formation assay (Figure 8F), suggesting that a reduction in the amount of ATP could attenuate the cisplatin-induced drug resistance in B16-F10 melanoma cells.

To delineate whether the phenomena of giant cells formation is unique to mouse melanoma, it was shown that cisplatin treatment also led to giant cells formation in two human melanoma RPMI7951 and C32TG cells (Figure S2B). Therefore, the formation of enlarged giant cells could be observed in mice as well as human melanoma cells after cisplatin treatment.

## 3. Discussion

Our data provide evidence that surviving melanoma cells can transform into giant cells in response to cisplatin treatment in vivo and in vitro and describe sufficient characteristics of giant cells for histopathologic examination. Because very few studies are involved in the formation of giant cells in melanoma, limited information is available regarding their clinical significance and generating mechanism. A similar observation in the clinic reported that biopsy specimens from 13 melanoma cases contained a high amount of “pleomorphic cells” (also termed “monster cells”) that exhibited a significantly enlarged nucleus and cytoplasm and that these cells with histopathologic features of malignancy were highly associated with ulceration, the depth of invasion, and the presence of multinucleated giant cells [30]. The histological examination of an additional malignant melanoma case reported a significantly high content of monster cells that expressed S100, which suggests that these monster cells can be a very aggressive variant of melanoma cells [31]. Another similar study reported that polyploid giant cancer cells existed in several ovarian cancer cell lines with the features of cancer stem-like cells and were resistant to cisplatin [32]. From our genomic DNA analysis by flowcytometry and cellular senescence maker staining (Figure S2A), the cisplatin-induced giant cells in melanoma were different from polyploid giant cancer cells in ovarian cancer and were not stress-induced senescent cells.

In our in vitro study, we defined cisplatin-induced giant cells by the cell surface area, and found that 3 µM cisplatin induced about 83% of the population to become giant cells (Figure 2E). When we detected the variation in the cell volume by flow cytometry, approximately 34.5% of the population became enlarged under the same conditions (Figure 3C). In parallel, we observed that cisplatin leads to a reduction in the thickness of the nuclei and the cytoplasm of B16-F10 cells (Figure 3B). This result implies that cisplatin treatment induces a certain proportion of B16-F10 cells to preferentially become flatter and thus increase their surface area but not their cellular volume. However, the analysis of the FSC by flow cytometry also supported the cisplatin-induced formation of giant cells. Since the giant cells with the morphology of senescence-like cells but without presence of SA-gal activity. One of the characteristics of senescent cells is irreversible cellular growth arrest. In our study, the cisplatin-induced giant cells retained the ability for cell divisions, and the Supplementary Materials Video S3 also provided the evidence for cell division of giant cells.

In addition to the abovementioned morphological changes, our results also demonstrated that the cisplatin-induced giant cells are more malignant because of the overexpression of S100, CD133, and ABCB5 in these giant cells. S100, an intermediate-filament protein, interacts with many cytoskeletal proteins that are involved in the control of the cytoskeleton assembly [33]. We speculate that S100 may play a role in the regulation of the cytoskeleton assembly during the formation of the giant cells. ABCB5 and CD133 have been proposed to be stem cell markers on the sentinel lymph nodes of melanoma patients [34]. ABCB5 is commonly overexpressed on circulating melanoma tumor cells [35]. ABCB5 mediates doxorubicin chemoresistance in human malignant melanoma, and is preferentially expressed on chemoresistant CD133-expressing tumor cells which indicates that this ABC transporter may be a marker of more primitive melanoma cells [22]. Similarly, our results show that the expressions of ABCB5 and CD133 were highly correlated in the giant cells, which also maintain the expression of the potential maker Ki-67 after cisplatin treatment (Figure 5 and Figure S1). In this study, we proposed a hypothesis that cisplatin-induced giant cells increase their surface area by flattening their shape to provide an increased membrane area for the assembly of a drug transporter, such as ABCB5. Hence, the giant cells are flatter and more drug-resistant than the untreated cells and are cross-resistant to doxorubicin and actinomycin D (Figure 6D).

Our data show that the giant cells contain a large number of mitochondria with a high membrane potential and have a high capacity to synthesize mitochondria (Figure 7). Besides, a high amount of energy seems required for the formation of giant cells (Figure 8), and such high energy that is produced in giant cells also provides adequate ATP for the efficient removal of the drug through the ABCB5 transporter. Moreover, we presumed that the motility of these cisplatin-induced giant cells was also decreased to conserve ATP (Figure 3G). It has been suggested that the mitochondria and the energy metabolism-related properties, including a high membrane potential of mitochondria, are novel indicators of lung cancer stem cells [36,37]. We observed that the giant cells had a higher capacity of drug efflux than the non-giant cells, which explains why cisplatin-induced giant cells were more resistant to chemotherapy drugs (Figure 6). ABCB5 functions, at least in part, as a drug-resistance mediator in the giant cells. We demonstrated that 2-DG attenuates the formation of giant cells and the expressions of ABCB5 and CD133 (Figure 8). In addition, 2-DG has been applied in combination with other therapies in human clinical trial [29].

The formation of giant cells is also found in human melanoma cells, although the cisplatin-induced percentages of giant cells in the tested human cell lines were lower than the percentages observed in the B16-F10 cell line. Moreover, the B16-F10 cells were selected from murine B16 melanoma cells by their metastasis ability and were more malignant than their parental B16-F0 cells [38]. The potential formation of giant cells may be associated with the malignancy degree of the melanoma cells.

## 4. Materials and Methods

### 4.1. Reagents and Antibodies

Unless otherwise indicated, all of the chemicals were purchased from Sigma (St. Louis, MO, USA). All of antibodies were stored at 4 °C until use: rabbit anti-S100 antibody (Dako Cytomation, Denmark), mouse anti-COX IV antibody (Abcam, Cambridge, UK), goat anti-ABCB5 antibody (Abnova, Taipei, Taiwan), Alexa-488-conjugated and Alexa-546-conjugated secondary antibodies (Molecular Probe, Eugene, OR, USA), rabbit anti-CD133 antibody, mouse anti-Ki-67 antibody, horseradish peroxidase-(HRP)-conjugated anti-mouse IgG antibody, HRP-conjugated anti-rabbit IgG antibody and HRP-conjugated anti-goat IgG antibody (Santa Cruz, CA, USA).

### 4.2. Cell Culture

All of cell lines were from American Type Culture Collection (ATCC). Murine melanoma cells, B16-F10 cells were cultured in Dulbecco’s modified Eagle’s medium (Invitrogen GIBCO, Carlsbad, CA, USA) with 10% fetal bovine serum (FBS; HyClone, Thermo Scientific, Logan, UT, USA), 2 mM glutamine, 10 µg/mL streptomycin, and 100 U/mL penicillin. All of the cultures were maintained in a humidified atmosphere containing 5% CO_2_ at 37 °C.

### 4.3. Tumor Growth In Vivo

All of the animal experiments were performed in accordance with the guidelines of National Sun Yat-Sen University approved by the Institutional Animal Care and Use Committee of the National Sun Yat-Sen University (approved number is 10005, 04/03/2011). Male C57BL/6 mice (6–8 weeks old; The Animal Center of the National Science Council, Taipei, Taiwan) were acclimated and caged in groups of four or less. All of the mice were maintained in a 12 h light/dark cycle and received drinking water and standard chow ad libitum. The C57BL/6 mice were subcutaneously injected with B16-F10 cells (5 × 10^5^ cells in 0.1 mL of phosphate-buffered saline (PBS) per mice). Ten days after the injection, the mice were administered by i.p. equal volumes of either PBS or 4 mg/kg cisplatin for three consecutive days each week (on the 10th, 11th, 12th, 17th, 18th, and 19th day after transplantation of melanoma cells). After 2-week administration of cisplatin, the mice were sacrificed by intraperitoneal injection of 100 mg/kg pentobarbital, and the tumors were dissected for HE staining and immunohistochemistry analysis. Tumor volumes were measured with a dial caliper and calculated using the following formula: volume (mm^3^) = width^2^ × length × 0.52.

### 4.4. Tissue Staining and Cellular Area Measurement

The processes of fix, and hematoxylin staining for melanoma tissue, were performed as described previously [39]. Sections were analyzed with a microscope (Leica, Germany). The method that was used to measure the area of cell and the nucleus was described previously [40]. The cell and nuclear areas of at least 500 individual cells were determined using the Image-Pro Plus software. For the immunohistochemistry study, the deparaffinized sections were subsequently incubated with the indicated primary antibody at 4 °C overnight. After the sections were washed three times with PBS, the cells were incubated with Alexa-488-conjugated (or Alexa-546-conjugated) secondary antibodies (1:1000 dilution). The cover slips with the treated cells were mounted on microscope slides.

### 4.5. Time-Lapse Video-Micrography for the Recording of the Formation of Giant Cells

The appropriate B16-F10 melanoma cells were seeded on a 12-well plate. The cells were maintained at 37 °C in a humidified 5% CO_2_ atmosphere in a stage heater, and the cell images were captured every 5 min. The digital images were obtained using a ZEISS LSM PASCAL multiphoton confocal microscope image system (LSM PASCAL; Carl Zeiss, Oberkochen, Germany).

### 4.6. Immunofluorescence and Cell Morphology Staining

B16-F10 cells were grown on glass coverslips. The cells were rinsed with PBS, fixed with 4% paraformaldehyde in phosphate buffer saline (PBS), and permeabilized with 0.1% Triton X-100 in PBS. To detect the cytoskeleton, the cells were stained with Alexa Fluor 488 phalloidin (Molecular Probes, Eugene, OR, USA) for 15 min at room temperature in the dark. The cells were then rinsed twice with PBS, incubated with 4′,6-diamidino-2-phenylindole (DAPI) in PBS for 5 min in dark, and then were rinsed twice with PBS. For immunofluorescence studies, the fixed and permeabilized cells were blocked with 3% bovine serum albumin in PBS for 30 min and subsequently incubated with the primary antibody overnight. The cells were then washed three times with PBS and then incubated with the corresponding Alexa-488-conjugated (or Alexa-546-conjugated) secondary antibody (1:1000 dilution; Molecular Probes, Eugene, OR, USA). Finally, the cells were rinsed twice with PBS and incubated with DAPI for 5 min. The fluorescent images of cells were captured using a confocal microscope image system (LSM PASCAL; Carl Zeiss, Oberkochen, Germany) or an inverted fluorescence Leica DM IL LED microscope (Leica, Germany). The cell and nuclear areas were analyzed using the Image-Pro Plus software. The analysis of 3D images were reconstituted from 25 confocal slices at 0.5 μm intervals using laser-scanning microscopy, and were further converted into staining intensity of actin filaments and DAPI by LSM PASCAL software (LSM PASCAL; Carl Zeiss, Oberkochen, Germany).

### 4.7. Mitochondrial Staining

B16-F10 cells were grown on glass coverslip. After the indicated drug treatment, the cells were incubated with 1 µM MitoTracker Red (Molecular Probes, Eugene, OR, USA) at 37 °C for 15 min and then washed with PBS. The cells were then fixed in 4% paraformaldehyde in cultured medium for 15 min and permeabilized with 0.1% Triton X-100 in PBS. To stain the actin filaments, the cells were stained with Alexa Fluor 488 phalloidin in PBS for 15 min at room temperature in the dark. To stain the nuclei, the cells were rinsed twice with PBS, incubated with DAPI for 5 min, and then rinsed twice with PBS. The fluorescence staining was viewed using a confocal microscope image system.

### 4.8. Mitochondrial Membrane Potential

B16-F10 cells were seeded onto glass slides at a density of 1 × 10^4^ cells per well in 12-well plates and allowed to reach exponential growth for 24 h before treatment. After 48-h treatment with cisplatin, the cells were incubated with 5,5′,6,6′-tetrachloro-1,1′,3,3′-tetraethylbenzimidazolylcarbocyanineiodide (JC-1; Fluka, Germany) or tetramethylrhodamine methyl ester (TMRM; Molecular Probes, Eugene, OR, USA). To measure the mitochondrial membrane potential by JC-1 staining, the cells were further incubated with 10 μg/mL JC-1 in PBS at 37 °C for 15 min. After the cells were washed with PBS, the images of the cells were recorded using confocal microscope. To detect the mitochondrial membrane potential by TMRM staining, the cells were incubated with 50 nM of TMRM in culture medium for 15 min. The cells were then resuspended in PBS, and their fluorescence intensities were measured using a FACSCalibur flow cytometer (BD Biosciences, San Jose, CA, USA).

### 4.9. Measurement of Intracellular ATP Content

The intracellular ATP of the melanoma cells was measured through a luciferin-luciferase bioluminescence method using the ATP Bioluminescence Assay Kit CLS II (Roche, Mannheim, Germany) following the manufacturer’s recommended protocol. The cisplatin-treated cells were washed, collected in ice-cold PBS, and adjusted to a concentration of 1 × 10^5^ cells/mL. Subsequently, the luciferase activities of the ATP production were determined using a luminometer (Berthold Detection Systems Orion II; Berthold Technologies, Bad Wildbad, Germany) and normalized to the cell numbers. A standard curve was generated using the known concentrations of ATP that were supplied with the kit and was used to calculate the concentration of ATP in each sample.

### 4.10. Western Blot Analysis

The cellular lysates from B16-F10 cells were extracted using a RIPA buffer containing 150 mM NaCl, 50 mM HEPES pH 7, 1% Triton X-100, 10% glycerol, 1.5 mM MgCl_2_, 1 mM EGTA, and protease inhibitors (Roche, Mannheim, Germany). The processes of Western blot including gel electrophoresis, transfer, and immunoband detection was performed as described previously [41]. The immunoband intensities were quantified by densitometric scanning. The corresponding values for immunoband intensities were normalized with respect to the immunoband intensities of β-actin, in the cells that were treated with PBS (control).

### 4.11. Quantitative Reverse-Transcription Polymerase Chain Reaction (qRT-PCR)

The processes of RNA extraction, reverse transcription, amplification, and detection of qRT-PCR were analyzed as described previously [39] by using the ABI Prism 7700 instrument. The products of the reverse transcription were amplified using ABCB5-specific primers (forward primer, 5′-TTCACAGTAGCCAGAGGAGC-3′; reverse primer, 5′-TTGCCACTGCCACTGGGA-3′), and CD133-specific primers (forward primer, 5′-AGCTCCCATCAGTGGATAGAGAA-3′; reverse primers, 5′-GGAATACTTTGGCTCATGTCCT-3′). The β-actin-specific primers (forward primer, 5′-TCACCCACACTGTGCCCATCTACGA-3′; reverse primer (5′-CAGCGGAAC CGCTCATTGCCAATGG-3′) were utilized as an internal control.

### 4.12. Flow Cytometric Analysis of Protein Expression, Multidrug Resistance (MDR), and Cell Size

B16-F10 cells were harvested, fixed with 4% paraformaldehyde in PBS, and then permeabilized with 0.1% Triton X-100 in PBS for 30 s. After blocking with 10% bovine serum for 30 min, the cells were incubated with anti-ABCB5 antibody and either anti-CD133 or anti-Ki-67 antibodies and then with Alexa-488-conjugated or Alexa-546-conjugated secondary antibodies. After the cells were washed with PBS, the dual-color fluorescence was acquired through the emission at the FL1 (Alexa-488) and FL2 (Alexa-546) spectra on a FACSCalibur flow cytometer (BD Biosciences, San Jose, CA, USA). The Vybrant^TM^ multidrug resistance assay kit (Molecular Probes, Eugene, OR, USA) was used to analyze the drug resistance of cells [42]. The B16-F10 cells were suspended in a tube and subsequently incubated with calcein AM for 15 min. The cells were then harvested on ice and immediately analyzed using the flow cytometer. For the cell size analysis, the size of the cells was recorded using the forward scatter (FSC).

### 4.13. Colony Formation Assays

B16-F10 cells were seeded as single-cell suspensions at 500 cells per well in 6-well plates overnight and then treated with cisplatin or 2-DG. After 7 days, the media was aspirated, and the plates were washed with PBS and stained and fixed with 1% crystal violet solution containing 4% paraformaldehyde in PBS for 20 min at room temperature. The plates were subsequently washed gently with water and dried.

### 4.14. Statistical Analysis

The tumor volume values are expressed as the means ± SEM. All other values are expressed as the means ± SD from three independent experiments. A *t*-test was applied to compare the differences between the two groups. When the data failed normality test, it was analyzed by a Mann-Whitney U test, and a difference was considered significant if *p* < 0.05. The statistical software package of SigmaStat (version 3.5, Systat Software, San Jose, CA, USA) was used for all of the statistical analysis.

## 5. Conclusions

In conclusion, we proved that melanoma cells can transform into giant cells that are more multiple drug resistant in response to cisplatin treatment, and such data are useful for the pathological examination of drug resistance and malignancy in melanoma. Furthermore, our evidence that blocking ATP synthesis can reduce the formation of giant cells provides a novel concept that can be used to design therapeutics for patients with melanoma.

## Supplementary Materials

The following are available online at https://www.mdpi.com/1422-0067/21/21/7892/s1, Figure S1: Giant cells have the ability to divide in vivo and in vitro. (A) The cellular Ki-67 in tissue specimens was immunostained with anti-Ki-67 antibody and Alexa-488-conjugated secondary antibody (green), and the nuclei were stained with DAPI (blue) in parallel. The images were captured by using a confocal microscope. Scale bar, 50 µm. (B) After 48 h treatment with 3 µM cisplatin, the division of the giant cells was captured by time-lapse video micrography. Red arrows indicate the mitotic cell. Scale bar, 50 µm. (C) The mitotic giant cells appeared in a tumor specimen from cisplatin-treated mice that was stained with HE. Red arrows indicate the mitotic cell. Scale bar, 20 µm. Figure S2: The senescence-associated β-gal staining image of B16-F10 and Human melanoma giant cells image. (A)The senescence-associated β-gal staining image of B16-F10 cells after cisplatin treatment. B16-F10 cells were treated with or without 3 μM cisplatin, and then SA-gal staining was investigated following a commercial kit protocol at indicated time. Only low percentage of SA-β gal positive giant cells can be observed at 6th day after cisplatin treatment. (B) The Human melanoma cell lines (RPMI7951 and C32TG) were treated with 1 µM cisplatin for 48 h, fixed, and then stained with phalloidin (green) and DAPI (blue). Scale bar, 50 µm. Figure S3: The viabilities of B16-F10 cells that were obtained from the MTT test and the cell number count were different. B16-F10 cells were cultured in 12-well plates. The cells were treated with different doses (0.1~10 µM) of cisplatin and then analyzed using the different methods. (A) The growth of B16-F10 cells was suppressed after cisplatin treatment. The cell number was counted at the indicated time points. (B) The viabilities of B16-F10 cells were analyzed using the MTT test after 48 h treatment with various doses of cisplatin. (C) The viabilities of the B16-F10 cells were analyzed by counting the cell numbers after 48 h exposure to various doses of cisplatin. The numbers of viable and dead cells were determined by trypan blue exclusion and counted using a hemocytometer. Video S1: The progression of control B16-F10 cell growth captured by time lapse recording. B16-F10 cells were treated with PBS (control), and then morphological changes were record by time-lapse confocal microscopy (LSM PASCAL; Carl Zeiss, Germany). Frames were taken every five minutes for 60h. Video S2: The progression of cisplatin-induced giant cells formation captured by time lapse recording. B16-F10 cells were treated with 3 μM cisplatin, and then morphological changes were record by time-lapse confocal microscopy (LSM PASCAL; Carl Zeiss, Germany). Frames were taken every five minutes for 60h. Video S3. The evidence for division of giant cells by time lapse recording. B16-F10 cells were treated with 3 μM cisplatin, and then morphological changes were record by time-lapse confocal microscopy (LSM PASCAL; Carl Zeiss, Germany). Frames were taken every five minutes for 10h.

## Abbreviations

ABC	ATP-binding cassette
ABCB5	ATP-binding cassette, sub-family B (MDR/TAP), member 5
FSC	forward scatter
MDR	multidrug resistance
P-gp	P-glycoprotein
SA-gal	senescence associated-β galactosidase
SSC	side scatter
