# Supplementary material for: Cisplatin-Induced Giant Cells Formation Is Involved in Chemoresistance of Melanoma Cells
Source: Int J Mol Sci. 2020 Oct 24;21(21):7892. doi: 10.3390/ijms21217892 (PMC7660656; doi:10.3390/ijms21217892)
Supplement: Supplementary file 1 [file ijms-21-07892-s001.zip › ijms-963774-supplementary/IJMS supplementary proof.doc]

**Supplementary Materials**

**Figure S1.** Giant cells have the ability to divide in vivo and in vitro. (A) The cellular Ki-67 in tissue specimens was immunostained with anti-Ki-67 antibody and Alexa-488-conjugated secondary antibody (green), and the nuclei were stained with DAPI (blue) in parallel. The images were captured by using a confocal microscope. Scale bar, 50 µm. (B) After 48 h treatment with 3 µM cisplatin, the division of the giant cells was captured by time-lapse video micrography. Red arrows indicate the mitotic cell. Scale bar, 50 µm. (C) The mitotic giant cells appeared in a tumor specimen from cisplatin-treated mice that was stained with HE. Red arrows indicate the mitotic cell. Scale bar, 20 µm.

**Figure S2.** The senescence-associated β-gal staining image of B16-F10 and Human melanoma giant cells image. (A)The senescence-associated β-gal staining image of B16-F10 cells after cisplatin treatment. B16-F10 cells were treated with or without 3 μM cisplatin, and then SA-gal staining was investigated following a commercial kit protocol at indicated time. Only low percentage of SA-β gal positive giant cells can be observed at 6th day after cisplatin treatment. Scale bar, 50 µm. (B) The Human melanoma cell lines (RPMI7951 and C32TG) were treated with 1 µM cisplatin for 48 h, fixed, and then stained with phalloidin (green) and DAPI (blue). Scale bar, 50 µm.

**Figure S3.** The viabilities of B16-F10 cells that were obtained from the MTT test and the cell number count were different. B16-F10 cells were cultured in 12-well plates. The cells were treated with different doses (0.1~10 µM) of cisplatin and then analyzed using the different methods. (A) The growth of B16-F10 cells was suppressed after cisplatin treatment. The cell number was counted at the indicated time points. (B) The viabilities of B16-F10 cells were analyzed using the MTT test after 48 h treatment with various doses of cisplatin. (C) The viabilities of the B16-F10 cells were analyzed by counting the cell numbers after 48 h exposure to various doses of cisplatin. The numbers of viable and dead cells were determined by trypan blue exclusion and counted using a hemocytometer.

**Video S1.** The progression of control B16-F10 cell growth captured by time lapse recording. B16-F10 cells were treated with PBS (control), and then morphological changes were record by time-lapse confocal microscopy (LSM PASCAL; Carl Zeiss, Germany). Frames were taken every five minutes for 60h.

**Video S2.** The progression of cisplatin-induced giant cells formation captured by time lapse recording. B16-F10 cells were treated with 3 μM cisplatin, and then morphological changes were record by time-lapse confocal microscopy (LSM PASCAL; Carl Zeiss, Germany). Frames were taken every five minutes for 60h.

**Video S3.** The evidence for division of giant cells by time lapse recording. B16-F10 cells were treated with 3 μM cisplatin, and then morphological changes were record by time-lapse confocal microscopy (LSM PASCAL; Carl Zeiss, Germany). Frames were taken every five minutes for 10h.

**Supplementary Materials and Methods**

***Cell culture***

All of cell lines were from American Type Culture Collection (ATCC). The RPMI 7951 and C32TG human melanoma cells were cultured in minimum essential medium (Invitrogen) with 10% FBS (HyClone) supplemented with 0.1 mM non-essential amino acids and 1 mM sodium pyruvate; the C32TG cell line was additionally supplemented with 0.05 mM 6-thioguanine. All of the cultures were maintained in a humidified atmosphere containing 5% CO_2_ at 37°C.

***Senescence-associated β-gal staining***

B16-F10 cells were treated with or without cisplatin. At the indicated time, the degree of cellular senescence was detected by senescence-associated β-galactosidase (SA-β-gal) assay using Cellular Senescence Assay Kit KAA002 (Chemicon). Cells were incubated in SA-β-gal Detection Solution for 12 h. Blue stained cells (senescent cells) were recorded under phase contrast microscopy.
